# Antimicrobial properties of green synthesized silver and chitosan nanocomposites

**DOI:** 10.6026/97320630019745

**Published:** 2023-06-30

**Authors:** Dhevishri S, B Devi Parameswari, Hariharan Annapoorni, MS Sathya Shankar, Rajesh Kumar S

**Affiliations:** 1 Department of Prosthodontics, MAHER, Chennai, India; 2 Department of Prosthodontics, MAHER, Chennai, India; 3 Department of Prosthodontics, MAHER, Chennai, India; 4 Department of Prosthodontics, MAHER, Chennai, India; 5 Department of Pharmacology, Saveetha dental college, SIMATS, Chennai, India

**Keywords:** Nanoparticles, silver, chitosan, green synthesis, antimicrobial, nano-composites

## Abstract

An eco-friendly and simple approach was carried out for the synthesis of silver-chitosan nanocomposites using *Azadirachta indica* and fluconazole-mediated aqueous extract. This extract acted as a reducing agent as well as a capping agent for the green synthesis of silver nanoparticles. Chitosan nanoparticles on the other hand were synthesized from the deacetylation of the chitin matrix. To confirm the nanoparticle synthesis, a UV- A visible spectrophotometer was used and FTIR analysis confirmed the presence of functional groups in the prepared extract. The morphological characteristics of silver and chitosan nanoparticles and as nano-composites were studied and confirmed using scanning electron microscopy (SEM) analysis. The synthesized silver-chitosan nanocomposites were subjected to well-loaded agar plates for the evaluation of antibacterial properties against the *Streptococcus mutans* and *Candida albicans* for their antifungal properties. The synthesized silver and chitosan nanoparticles showed antibacterial and antifungal activities against common oral micro flora such as *Streptococcus mutans* and *Candida albicans* which were measured using the zone of inhibition method. This approach is a one-step, economical and eco-friendly, biocompatible, and effective alternative for nanoparticle synthesis for various prosthetic applications.

## Background:

Green synthesis is increasingly in demand and needs, its time-conservative approach under simple conditions in laboratories. Silver nanoparticles being proven as an effective anti-inflammatory, antibacterial, and antifungal agent need is widely expanding in the medical and dental fields. [1] Nano-silver synthesis has been carried out in a number of ways like chemical reduction, and ion sputtering and their readily available commercial forms are still used in and out in the daily research fields. [2] However, the use of chemicals and high energy requirements for synthesis stand as an environmental hazard for many years, which led the way to the greener approach of silver nanoparticle synthesis. Green synthesis of silver nanoparticles is becoming an effective procedure, proving their importance in medicine and dentistry. Green synthesis employs plant extracts, sugars, polymers, and even microorganisms as reducing and capping agents for nano-synthesis. This approach also provides the advantages of being simple and easily reproducible and they are known to produce stable by-products. Green synthesis using plant extracts is relatively faster in preparation time and a lot in this field is still not explored to its full extent. [3] Plant extracts from beetroot, *Solanum tricobatum*, marigold flower, *Melia dubia*, and leaf extract of *Acalypha indica* are some of these reported in the earlier studies with nanoparticle synthesis. [4] Likely, this study utilizes the leaf extract of *Azadirachta indica* (neem plant) form the family of Meliaceae was used along with fluconazole (antifungal agent) as a reducing agent for the synthesis of silver nanoparticles. Silver nanoparticles (Ag-NPs) can be produced from plant leaf extract without the need of any chemical or physical constituents; however, fluconazole was added to the plant extract to investigate if there is an effective increase of antimicrobial activity against the common oral pathogens *Streptococcus mutans* and *Candida albicans*. Chitosan nanoparticles (CS-NPs) are derived from natural biopolymer by deacetylation of chitin. [5] These are known for their various biocompatible and physicochemical properties. However, studies conducted on silver-chitosan as nanocomposites for evaluating their antimicrobial properties in the literature are scarce. This study highlights a greener approach for the synthesis of silver, and chitosan nanoparticles individually and their nanocomposites as antimicrobial agents against common oral pathogens.

##  Materials and Methods:

Green synthesis of any nanoparticle is obtained from an aqueous extract by reducing a metal salt from an aqueous solution at room temperature within a few minutes to hours.

## Preparation of plant extract:

A. indica dried leaves powder (Nisarg organic Farm, India) was commercially available and they were diluted in distilled water and 5ml of fluconazole was added to this solution. The prepared solution was boiled for 30 minutes for any contaminants, passed through Whatman's filter paper twice, and filtered. The prepared plant extract was stored at 4-5 °C for further characterization analysis.

## Green synthesis of silver nanoparticles:

Silver nitrate GR was commercially purchased (Merck, India). Erlenmeyer flask was used to prepare the silver nitrate solution of 100mL, 1mM in concentration. Then 1, 2 and 3mL of plant extract was added to 10mL of silver nitrate solution. This colloidal solution was kept in a dark chamber at room temperature for reduction of silver ions. The reduction of silver ions was confirmed by the solution's visible color change from clear colorless to brown.

##  Green synthesis of chitosan nanoparticles:

A total of 5g of chitin powder was commercially purchased and added to 20 ml of deionized water. The mixed solution was sonicated for 20 minutes and then dried. This sonicated sample was mixed with 50% sodium hydroxide and heated at 90C for 80 minutes. The heated mixture was filtered and the residue was washed and dried in a hot air oven. The prepared residue was mixed with amine (aromatic) 2.0mmol solution and with water 20ml. This chitin extract was subjected to a magnetic stirrer for 3 -4 hours.

##  Characterization of synthesized silver and chitosan nanoparticles:

## UV- Vis Spectral analysis:

UV - visible spectrophotometer (UV-1800, Japan) with a resolution of 1nm between the range of 200 to 800nm was utilized. X-axis represents the wavelength (nm) and Y-axis depicts the absorbance. The reduction of silver ions was initiated by the biocomponents of the Azadiracta indica plant extract. The AgNPs from Azadiracta indica were observed at 430 nm which confirms the nanoparticle synthesis.

##  FT-IR analysis:

The plant extract has a dual role in nanoparticle synthesis namely as a reducing as well as a capping agent. The FT-IR analysis is used to confirm the presence of functional groups in the silver nanoparticle. Buffer-subtracted transmission spectra were noted and recorded from the range of 600 - 3500 cm-1. This value was obtained using a Perkin Elmer spectrum 100 FTIR spectrometer confirming the surface structure and changes in the structure of modified proteins (MG) present in various concentrations of AgNPs.

## Preparation of Ag-Ch nanocomposites

The synthesized AgNPs were added to the chitosan nanoparticles solution in three varying ratios namely 1: 1, 1:3, and 3:1 of Ag: Chitosan nanocomposites. After adding the nano-silver pellets with a chitosan solution in varying ratios, there were subjected to a magnetic stirrer for 3- 4 hours to obtain a homogeneous solution.

## Characterisation of Ag-Ch nanocomposites using SEM analysis

The surface morphology of the prepared nanocomposites (Ag - Ch) was studied through Scanning electron microscopy (SEM). The SEM images show the spherical silver nanoparticles distributed among the clusters of chitosan nanoparticles, confirming the nanocomposite presence in the colloidal solution.

## Antimicrobial analysis of Ag-Ch nanocomposites

**Figure 1:**
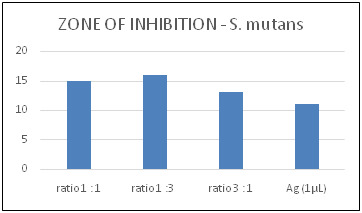
Zone of inhibition of prepared nanocomposites against the *S. mutans* species when compared with synthesized nano silver.

**Figure 2:**
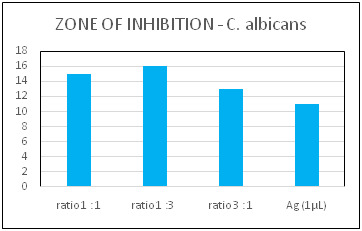
Zone of inhibition of prepared nanocomposites against *C. albicans* species when compared with synthesized nano-silver.

In the present study, two common oral microorganisms were cultured and studied for the zone of inhibition around these synthesized nanocomposites namely, *Streptococcus mutans* (gram-negative) bacteria and *Candida albicans* (fungal species) Antibacterial and Antifungal activities of Ag - Chitosan nanocomposites were observed by well - diffusion method. Blood agar served as the medium for microbial activation, which was taken in culture plates. Four wells were made, of which nano-silver with three varying ratios of nanocomposites. These well loaded agar plates were incubated at 36°C for 24 hours. After incubation, the zone of inhibition around the well was observed; this denotes the antimicrobial activities of Ag and chitosan nanoparticles individually and in combination (Figure 1 and Figure 2).

## Results:

The zone of inhibition (mm) for both *Candida albicans* and *Streptococcus mutans*f as antibacterial and antifungal activities of three varying combinations of nanocomposites l where measured and noted, which were then entered in a Microsoft Excel spreadsheet and analyzed using SPSS Software [Statistical Software Package for Social Sciences - version 2. 1] (Table 1). Table 1 lists the antibacterial and antifungal activities of Ag-Ch nanocomposites in varying concentrations compared with nano-silver of 1µL in concentration.

The zone of inhibition (ZOI) of Ag-Ch nanocomposites is demonstrated against bacterial and fungal cultures. The highest ZOI against the *S. mutans* species was displayed when nano silver and chitosan are in equal proportions (Figure 1). However, chitosan in three parts to one part of nano-silver in ratio has shown the highest ZOI against *C. albicans.* (Figure 2) Ag and Ch Nps exhibit their antimicrobial potential through their mechanism of adhesion penetration into the cell membrane of these microbial cells. These nanoparticles possess the ability of free radical production against these microbes, thereby, inhibiting their growth in these matrices.

## Discussion:

In this study, silver (Ag) and chitosan nanoparticles are chosen due to their proven antimicrobial efficiency and higher biocompatibility. Silver nanoparticles have proven to be effective against a broad spectrum of oral bacterial and fungal species from the existing literature. However, chitosan has modifiable functional groups, which promote their higher drug-delivering capacity. Mariselvam *et al.* 2014, Zhang *et al.* in 2010, and Panigrahi in 2013 followed the green synthesis approach in silver nanoparticles synthesis from plant leaf extract, root, fruit, latex, bark, bacteria, and enzymes in their studies. [6 7] Ahmed *et al.* in 2015 concluded that plant extracts are the best capping material for silver nanoparticle synthesis, hence in this study silver and chitosan nanocomposites are synthesized from *Azadirachta indica* species (neem leaf extract). [8] These species are well known for their antibacterial and antifungal activities against many common oral pathogens. Banerjee et al 2014 concluded that aqueous neem leaf extract acts as a reducing agent by reducing silver nitrate into silver salts, which is in accordance with this study. [9] Whereas, in this in -vitro study the green synthesized silver nanoparticles were obtained from *Azadirachta indica* (neem leaves) extract combined with Fluconazole, a commercially available antifungal agent. In this study, the green synthesized silver nanoparticles are analysed and confirmed through UV-vis spectrophotometry, like the existing studies in the literature such as Dugganaboyana *et al.* (2017), Mousavi *et al.* (2019 and 2020), Rajeshkumar *et al.* (2021) [10,11, 12,13]. These green synthesized silver and chitosan nanoparticles were confirmed using this method, indicating the wavelength between 420 -455nm in range. This study has also confirmed the surface characteristics of synthesized nanoparticles either alone or in combination.

## Conclusion:

The findings of this study conclude that green-synthesized silver and chitosan nanocomposites are viable alternatives for nanoparticle synthesis and these green-synthesized nanocomposites are potential antibacterial and antifungal agents against the two commonly detected oral micro-organisms. This study also raises the scope for further investigations on different oral pathogens against these nano-composites.

## Figures and Tables

**Table 1:** Ag-Ch nanocomposites zone of inhibition (in mm)

**ZONE OF INHIBITION (mm)**	**(Ag-Ch)1:1**	**(Ag-Ch)1:3**	**(Ag-Ch)3:1**	**Ag(1µL)**
*S. MUTANS*	33	30	26	23
*C. ALBICANS*	15	16	13	11
